# Delayed splenic rupture following video-assisted thoracoscopic left upper lobectomy: a case report and literature review

**DOI:** 10.3389/fsurg.2026.1853919

**Published:** 2026-06-17

**Authors:** Wei Li, Huawen Hu, Zhuang Cheng, Erping Xi

**Affiliations:** Department of Cardiothoracic Surgery, General Hospital of Central Theater Command of Chinese People’s Liberation Army, Wuhan, China

**Keywords:** case report, literature review, lung lobectomy, non-small cell lung cancer, postoperative complication, splenic rupture, video-assisted thoracoscopic surgery

## Abstract

Video-assisted thoracoscopic surgery (VATS) is the preferred surgical approach for early-stage lung cancer, though it can rarely cause life-threatening delayed splenic rupture (DSR). With an insidious latent course, DSR frequently eludes early diagnosis and may progress to fatal hemorrhagic shock. We herein describe a 70-year-old male with a prior subtotal gastrectomy who developed DSR on postoperative day 6 after VATS left upper lobectomy. The patient presented with acute hypotension and tachycardia without overt abdominal signs, was confirmed via bedside ultrasonography (US) and diagnostic peritoneal paracentesis (DPP), and recovered uneventfully after emergency splenectomy. A pooled analysis of nine cases revealed that VATS-related DSR predominantly complicates left-sided thoracic surgery, manifesting as hemodynamic instability and progressive hematological decline with subtle abdominal findings, which commonly leads to early misdiagnosis. Major etiologies include intraoperative transdiaphragmatic injury and postoperative diaphragmatic traction aggravated by abdominal adhesions. Contrast-enhanced computed tomography (CT) remains the diagnostic gold standard for stable patients, while bedside US and DPP enable prompt emergency diagnosis. Individualized management is tailored to hemodynamic status and splenic injury severity. Meticulous intraoperative technique, rigorous postoperative monitoring, and high clinical vigilance, particularly for patients with abdominal adhesions, are critical to prevent DSR, reduce diagnostic delay, and optimize surgical outcomes.

## Introduction

Video-assisted thoracoscopic surgery (VATS) is the preferred surgical approach for curative resection of early-stage lung cancer. It provides multiple clinical benefits, including less intraoperative trauma, faster postoperative recovery, and superior clinical outcomes (1). Nevertheless, rare but severe complications may still occur, such as vascular injury, bronchopleural fistula, and diaphragmatic laceration. Splenic rupture, particularly delayed splenic rupture (DSR)—defined as splenic rupture occurring more than 48 hours after the initial traumatic insult (2)—represents a catastrophic complication following VATS. To date, fewer than 10 relevant cases have been reported. The prolonged latent period of DSR makes early diagnosis challenging, and delayed intervention can progress to fatal hemorrhagic shock (3). Herein, we report a case of DSR on postoperative day (POD) 6 following VATS left upper lobectomy (LUL) for non-small cell lung cancer (NSCLC). We also systematically reviewed published literature to discuss the clinical characteristics and management strategies of this complication, aiming to provide references for clinical practice.

## Case description

A 70-year-old male was admitted to our hospital following the detection of a left upper lobe pulmonary mass on routine chest computed tomography (CT) screening. Percutaneous transthoracic needle biopsy and subsequent histopathological examination confirmed poorly differentiated NSCLC. The patient had a 20-pack-year smoking history and quit smoking 1 month prior to admission, with no history of alcohol consumption. Over 30 years earlier, he had undergone subtotal gastrectomy for a gastric ulcer. He denied any history of hepatitis, liver cirrhosis, schistosomiasis, or recent abdominal trauma. Preoperative abdominal ultrasonography (US) revealed multiple simple hepatic cysts (the largest measuring 24 × 20 mm), with normal liver function test results. No imaging features suggestive of hydatid disease or other pathological lesions were observed, and no splenic abnormalities including splenomegaly, subcapsular hematoma, or parenchymal injury were identified.

Ten days after admission, the patient underwent elective VATS-LUL. The surgical approach was via a 4-cm incision in the 4th intercostal space at the midaxillary line and a 1.5-cm port at the posterior axillary line of the 7th intercostal space. The operation was completed smoothly, with a duration of approximately 120 minutes and an estimated blood loss of 200 mL. Intraoperative vital signs were stable throughout the procedure, and no blood transfusion was administered.

The first 5 postoperative days were characterized by an uneventful recovery, with stable vital signs and gradual restoration of gastrointestinal function. On the morning of POD 6, the patient developed sudden hypotension (95/60 mmHg) and tachycardia (110 beats per minute), without chest pain, fever, or dyspnea. Suspecting postoperative hypovolemia, fluid resuscitation was promptly initiated. However, the blood pressure transiently improved before deteriorating again.

Emergency routine blood tests demonstrated markedly decreased hematological parameters, all of which were far below the lower reference limits: red blood cell (RBC) 1.79 × 10^12^/L, hemoglobin (Hb) 50 g/L, and hematocrit (Hct) 16.1%, compared with the values on POD 5 (RBC 3.43 × 10^12^/L, Hb 94 g/L, and Hct 30.8%). High clinical suspicion for active bleeding prompted a comprehensive physical examination, which revealed no positive signs of thoracic bleeding. Only mild abdominal distension and diminished bowel sounds were noted, without abdominal guarding, tenderness, or rebound tenderness.

**Figure 1:**
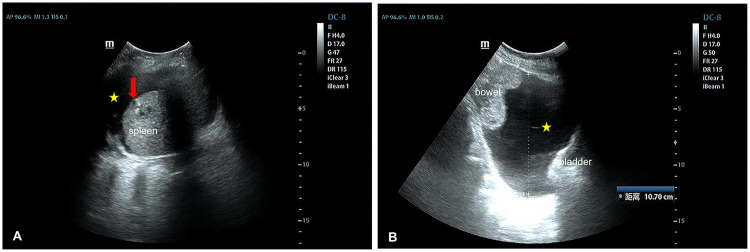
Abdominal ultrasonography reveals **(A)** discontinuity of the splenic capsule contour at the diaphragmatic surface with heterogeneous parenchymal echotexture (red arrow), and a significant perisplenic anechoic fluid collection (yellow star); **(B)** massive anechoic fluid accumulation in the lower abdominal cavity (yellow star).

**Figure 2:**
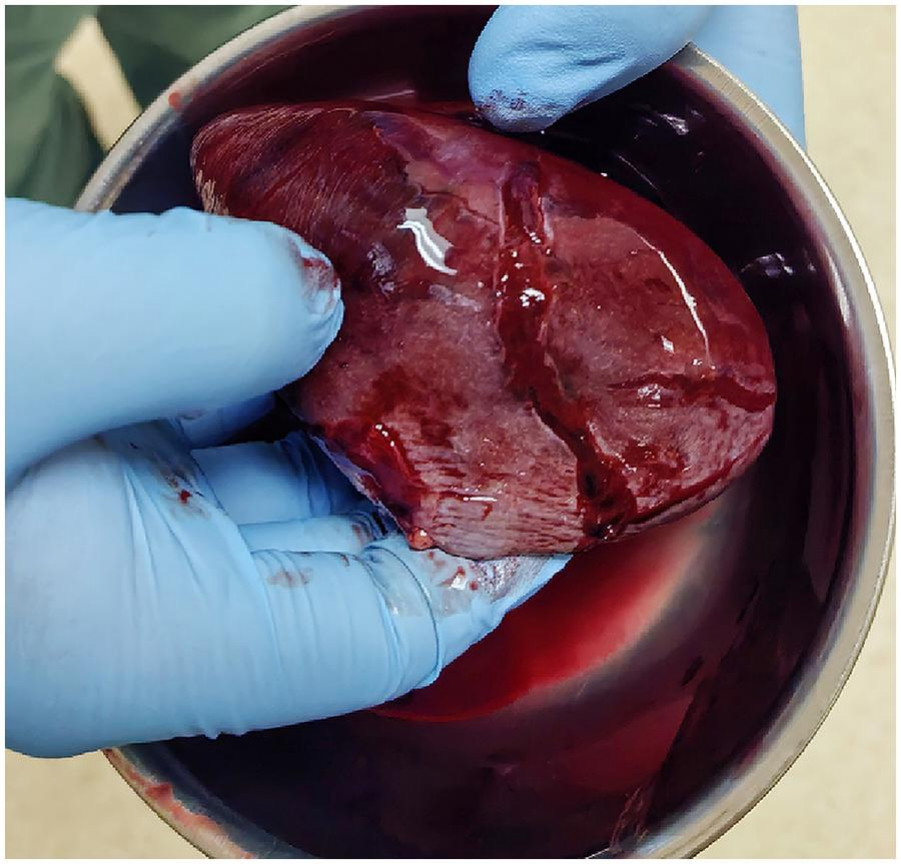
Gross inspection of the excised spleen shows two lacerations at the sites of splenic rupture.

Emergency abdominal US identified discontinuity of the splenic capsule and significant hemoperitoneum (Figure 1), which was confirmed by diagnostic paracentesis that aspirated non-clotting blood. A definite diagnosis of intra-abdominal hemorrhage (suspected splenic rupture) was made, and emergency laparotomy was performed urgently. Intraoperatively, two active bleeding splenic capsular lacerations were identified (Figure 2), with an estimated 2500 mL of hemoperitoneum. No injury to the diaphragm or other abdominal organs was observed. Due to uncontrollable bleeding and the patient's advanced age, splenectomy was performed. During the operation, 3 units of leukocyte-reduced packed RBCs, 600 mL of fresh frozen plasma and 500 mL of cell-salvaged autologous blood were administered. Postoperatively, the patient was transferred to the intensive care unit (ICU) for close monitoring and supportive treatment. A repeat routine blood test 2 hours postoperatively showed a marked improvement in hematological parameters: RBC 3.92 × 10^12^/L, Hb 109 g/L, and Hct 33.4%.

After 48 hours of ICU monitoring with stable vital signs and no further bleeding, the patient was transferred to the general ward. He had an uneventful recovery following splenectomy and was discharged on POD 14.

## Discussion

Splenic rupture following VATS is an extremely rare complication, with no reported incidence rate due to the limited number of cases. To further clarify the clinical characteristics of this complication, we systematically retrieved and summarized 8 previously published cases of post-VATS splenic rupture from the existing literature (4–11). Combined with the present case, a total of 9 cases were included in the pooled analysis (Table 1).

**Table 1:** Clinical characteristics of 9 cases of splenic rupture following video-assisted thoracoscopic surgery.

Case no.	Author	Gender	Age (y)	Past abdominal surgery	Thoracic procedure	Onset time	Core clinical manifestations	Diagnostic methods	Treatment	Prognosis	Postulated etiology
1	Zhang Z (4)	M	24	No	Left lower lobe lesion resection	POD 7	Abdominal distension/pain, mild abdominal tenderness	Abdominal US, abdominal CT	Splenectomy	Recovered	Excessive force on the left hemidiaphragm
2	Li J (5)	F	57	No	Left lower lobectomy	Intraoperative	Refractory hypotension, progressive Hb drop	FAST, diagnostic abdominal paracentesis	Splenorrhaphy	Recovered	Transdiaphragmatic blunt trauma
3	Yue H (6)	F	38	No	Left lower lobectomy	1 h postop	Sudden hypotension, Hb drop	Diagnostic abdominal paracentesis, abdominal CT	Splenorrhaphy	Recovered	Blunt trauma by surgical instruments
4	Flores RM (7)	M	57	N/A	Left upper lobe wedge resection	Postop	Hypotension, Hct drop	Abdominal CT	Splenectomy	Recovered	Surgical instrument blunt injury
5	Forti Parri SN (8)	N/A	51	N/A	Pleural mass resection	POD 2	Hb drop, mild abdominal tenderness	Abdominal CT	Conservative (angiographic splenic artery embolization)	Recovered	Transdiaphragmatic blunt trauma
6	Cohen JB (9)	M	82	Cholecystectomy	Left lower lobectomy	Intraoperative	Hypotension, Hb drop	Abdominal CT	Conservative (transfusion + vasoactive drugs)	Recovered	Left lung resection–related diaphragmatic elevation/tension, abdominal adhesions
7	Liu CT (10)	F	67	No	Left lower lobectomy	5 h postop	Hypotension, Hb drop, hypovolemia, abdominal distension/pain	Abdominal US, abdominal CT	Splenectomy	Recovered	Transdiaphragmatic blunt trauma
8	Hu X (11)	M	50	No	Left lower lobectomy	90 min postop	Tachycardia, refractory hypotension, hemorrhagic shock, Hb drop	E-FAST, ultrasound-guided paracentesis	Splenectomy	Recovered	Diaphragmatic traction-induced capsular tear or sudden increases in intra-abdominal pressure
9	Present case	M	70	Subtotal gastrectomy	Left upper lobectomy	POD 6	Sudden hypotension, tachycardia, severe Hb/Hct drop, abdominal distension, diminished bowel sounds	Abdominal US, diagnostic abdominal paracentesis	Splenectomy	Recovered	

POD, postoperative day; US, ultrasonography; CT, computed tomography; FAST, focused assessment with sonography for trauma; E-FAST, extended Fast; Hb, hemoglobin; Hct, hematocrit.

Pooled analysis of these 9 cases identified three core clinical features of post-VATS splenic rupture. First, it occurs predominantly following left-sided thoracic surgery, which is attributed to the anatomical proximity of the spleen to the left hemidiaphragm. Second, its onset time is variable, ranging from the intraoperative period to POD 7. Third, hemodynamic instability and progressive declines in hematological parameters are the dominant clinical manifestations, whereas abdominal symptoms are often subtle with no obvious positive signs, which renders it easily overlooked in the early stage.

Post-VATS splenic rupture, particularly DSR, presents considerable clinical diagnostic challenges. Most patients exhibit insidious early symptoms. Many only develop hemodynamic abnormalities (tachycardia and hypotension) and decreased hematological parameters (reduced Hb and Hct) without typical abdominal positive signs. These non-specific manifestations are predisposed to misdiagnosis as postoperative thoracic bleeding or hypovolemia of other etiologies.

In the present case, the patient developed hypotension and tachycardia on POD 6, accompanied by abdominal distension and diminished bowel sounds, but without abdominal guarding, tenderness, or rebound tenderness. This led to an initial misdiagnosis of uncomplicated postoperative hypovolemia. Therefore, for patients undergoing left-sided VATS, any unexplained perioperative hemodynamic instability and acute decline in Hb/Hct—even in the absence of typical abdominal positive signs—should prompt a high index of suspicion for splenic rupture, with timely diagnostic evaluation initiated promptly.

Abdominal US and CT are the two primary imaging modalities for the diagnosis of splenic rupture, with distinct advantages and applicable scenarios. Additionally, diagnostic peritoneal paracentesis (DPP) serves as a crucial emergency auxiliary diagnostic method, though it is no longer adopted as a routine standard procedure in tertiary and quaternary centres. US, including focused assessment with sonography for trauma (FAST) and extended FAST (E-FAST), serves as the first-line modality for emergency screening. It is rapid, noninvasive, repeatable, and does not require patient transport, making it ideal for hemodynamically unstable patients who cannot be transferred for further imaging (12). Nevertheless, US is inferior to CT in the accurate grading of splenic injury and fails to guide interventions like splenic artery embolization. Furthermore, compared with CT, US is more likely to underestimate or miss splenic injury adjacent to the diaphragm, and false-negative or indeterminate findings may hinder definitive diagnosis and delay targeted treatment (13, 14). CT can clearly demonstrate the extent of splenic injury and assess its severity, providing critical imaging evidence for surgical planning and robust guidance for conservative management or interventional embolization of mild splenic injury. Contrast-enhanced CT serves as the diagnostic gold standard for hemodynamically stable or stabilized trauma patients. However, CT must be readily available and be performed only in hemodynamically stable patients or those responsive to fluid resuscitation (3, 12, 13, 15). Despite the potential risk of injuring intra-abdominal organs, DPP is a specific confirmatory procedure that provides direct evidence of intra-abdominal hemorrhage and serves as an important auxiliary diagnostic method in emergency settings. Notably, ultrasound-guided DPP can substantially reduce the risk of accidental visceral injury (16, 17). The combination of these diagnostic approaches significantly improves the accuracy and timeliness of diagnosis, thereby avoiding delayed intervention.

In the present case, emergency DPP was prioritized over immediate abdominal CT for three critical clinical reasons. First, the elderly patient presented with persistent unstable hemodynamics (sustained hypotension and tachycardia). Bedside DPP rapidly confirmed massive intra-abdominal hemorrhage, avoiding delayed intervention and the risk of hemodynamic collapse caused by patient transfer for CT scanning. Second, progressive hypovolemia required urgent surgical intervention. At this critical stage, identifying active intra-abdominal bleeding was the primary diagnostic goal, whereas precise CT-based injury grading was non-urgent and would not change the surgical strategy. Third, DPP was performed to further verify bedside US findings. As US demonstrated significant hemoperitoneum, DPP near McBurney's point was deemed safe and feasible. Notably, DPP served as an emergency auxiliary measure for unstable critical conditions rather than a routine diagnostic method. For hemodynamically stable patients with suspected splenic rupture in our institution, contrast-enhanced abdominal CT remains the gold-standard examination.

The management of post-VATS splenic rupture follows an individualized strategy based on the patient's hemodynamic status and the severity of splenic injury. For patients with unstable hemodynamics, massive intra-abdominal hemorrhage or uncontrollable bleeding, and severe splenic injury, emergency surgical intervention is indicated promptly. Splenectomy is the primary surgical approach, particularly for elderly patients or those with comorbidities who cannot tolerate prolonged surgical procedures. For patients with stable hemodynamics and mild splenic injury, splenorrhaphy can be selected to preserve splenic immunological function (18). For patients with minimal bleeding and stable clinical conditions, conservative management such as angiographic splenic artery embolization, blood transfusion and supportive treatment can be adopted (19). During the treatment process, timely and effective blood transfusion and fluid resuscitation are critical to maintaining the patient's hemodynamic stability and improving the success rate of treatment. In the present case, splenectomy was performed considering the patient's advanced age and uncontrollable splenic bleeding. The patient achieved a good postoperative recovery, which verified the rationality of this treatment decision.

The etiology of post-VATS splenic rupture remains complex and not fully elucidated. Three major potential etiologies have been summarized from reported cases. First, transdiaphragmatic blunt trauma caused by surgical instruments and manipulation is the most commonly reported etiology (4–8, 10). The spleen lies in the left upper quadrant abdomen and is closely adjacent to the left hemidiaphragm. During left-sided VATS, excessive force, blind dissection, or improper instrument positioning may cause blunt trauma to the spleen, leading to capsular laceration and rupture even with an intact diaphragm. Second, indirect injury secondary to diaphragmatic elevation and increased tension has been proposed in 2 cases (9, 11). Following left pulmonary lobectomy, particularly LUL, the residual thoracic cavity volume increases significantly, leading to elevation and increased tension of the left hemidiaphragm. The elevated and tense diaphragm may exert traction on the splenic capsule via ligaments and/or adhesions, resulting in capsular laceration and rupture of the spleen. This risk is particularly prominent in patients with a history of abdominal surgery complicated by abdominal adhesions. Third, postoperative sudden increase in intra-abdominal pressure (e.g., sneezing, coughing, defecation) may induce splenic rupture (11). In the present case, the patient had a more than 30-year history of subtotal gastrectomy, which likely resulted in abdominal adhesions and fixed the spleen in position. During VATS-LUL, surgical instruments inserted through the 7th intercostal port may have caused occult transdiaphragmatic splenic injury and subsequent subcapsular hematoma formation. In the early postoperative period, limited patient activity resulted in mild diaphragmatic traction on the splenic capsule, keeping the subcapsular hematoma relatively stable. With gradual postoperative ambulation, the expanded residual thoracic cavity induced persistent elevation of the left hemidiaphragm. Excessive diaphragmatic traction on the splenic capsule ultimately led to DSR on POD 6.

Surgical manipulation is the primary risk factor for post-VATS splenic rupture. Standardized intraoperative and postoperative preventive measures are essential to minimize the incidence of this complication. Surgeons should possess a thorough understanding of thoracic anatomy, perform gentle manipulation, and standardize the positioning and handling of surgical instruments to reduce the risk of transdiaphragmatic blunt splenic injury during left-sided VATS. For patients with a history of abdominal surgery, careful attention should be paid to potential abdominal adhesions, and extra caution exercised to prevent diaphragmatic traction injury. Postoperatively, patients should be instructed to avoid activities that cause a sudden increase in intra-abdominal pressure, so as to reduce the risk of DSR.

## Conclusion

Splenic rupture following VATS is an extremely rare but life-threatening complication that occurs predominantly after left-sided thoracic surgery with variable onset. It typically presents with hemodynamic instability and decreased hematological parameters, while abdominal signs are often nonspecific or even absent, leading to a high risk of misdiagnosis. DSR is particularly insidious in onset and is more easily misdiagnosed in the early stage. Delayed diagnosis and treatment can further increase the risk of fatal hemorrhagic shock.

In conclusion, meticulous intraoperative surgical techniques during left-sided VATS, intensified postoperative monitoring of vital signs and hematological indicators, and maintaining a high clinical suspicion for splenic rupture are crucial for preventing this catastrophic complication, reducing misdiagnosis, and improving clinical outcomes.

## Data Availability

The original contributions presented in the study are included in the article/Supplementary Material, further inquiries can be directed to the corresponding authors.

## References

[1] Sihoe ADL. Video-assisted thoracoscopic surgery as the gold standard for lung cancer surgery. Respirology. (2020) 25 Suppl 2:49–60. 10.1111/resp.13920 32734596

[2] Schwartz SI Shires GT Spencer FC. Principles of Surgery. 5th ed New York, NY: McGraw-Hill Book Company (1989). p. 271–3.

[3] Kodikara S. Death due to hemorrhagic shock after delayed rupture of spleen: a rare phenomenon. Am J Forensic Med Pathol. (2009) 30(4):382–3. 10.1097/PAF.0b013e3181c03caf 19901812

[4] Zhang Z. A care report of splenic rupture with subcapsular hematoma after thoracoscopic surgery. Chin J Endosc. (2001) 5(7):27. 10.3969/j.issn.1007-1989.2001.05.064 (Article in Chinese).

[5] Li J Wang L. Anesthesia management for a patient with splenic rupture during thoracoscopic surgery. J Clin Anesthesiol. (2018) 34(3):312. 10.12089/jca.2018.03.026 (Article in Chinese).

[6] Yue H Wang J Zeng W Zhao W Dang X Zhang R. A successful case of splenic rupture with hemorrhagic shock after VATS. CMCE. (2025) 7(1):E684. 10.3760/cma.j.cmcr20250212-00480 (Article in Chinese).

[7] Flores RM Ihekweazu U Dycoco J Rizk NP Rusch VW Bains MS. Video-assisted thoracoscopic surgery (VATS) lobectomy: catastrophic intraoperative complications. J Thorac Cardiovasc Surg. (2011) 142(6):1412–7. 10.1016/j.jtcvs.2011.09.028 22014713

[8] Forti Parri SN Guiducci GM Domanico A Tugnoli G. Splenic rupture after videothoracoscopic procedure: an unusual complication conservatively managed. J Thorac Cardiovasc Surg. (2014) 148(5):e236–7. 10.1016/j.jtcvs.2014.08.004 25167983

[9] Cohen JB Hirschi MR Patel SY Liu J. Non-thoracic source of bleeding during left-sided thoracic surgery. Cureus. (2019) 11(5):e4593. 10.7759/cureus.4593 31309018 PMC6609276

[10] Liu CT Wu TT Ding YY Lin JL Zhou S Liu H. Case report: hemodynamic instability caused by splenic rupture during video-assisted thoracoscopic lobectomy. Front Surg. (2022) 9:900396. 10.3389/fsurg.2022.900396 35529913 PMC9073001

[11] Hu X Xu H Xie Y. Splenic rupture after video-assisted thoracoscopic lobectomy: a case report. J Surg Case Rep. (2025) 7:rjaf240. 10.1093/jscr/rjaf240 PMC12301181 40727070

[12] El-Matbouly M Jabbour G El-Menyar A Peralta R Abdelrahman H Zarour A. Blunt splenic trauma: assessment, management and outcomes. Surgeon. (2016) 14(1):52–8. 10.1016/j.surge.2015.08.001 26330367

[13] Coccolini F Montori G Catena F Kluger Y Biffl W Moore EE. Splenic trauma: wSES classification and guidelines for adult and pediatric patients. World J Emerg Surg. (2017) 12:40. 10.1186/s13017-017-0151-4 28828034 PMC5562999

[14] Vc A Yeli RK N H Sheelavant N. Role of USG and CT in the evaluation of abdominal trauma. Cureus. (2025) 17(11):e96943. 10.7759/cureus.96943 41409972 PMC12706502

[15] Hassan R Abd Aziz A Md Ralib AR Saat A. Computed tomography of blunt spleen injury: a pictorial review. Malays J Med Sci. (2011) 18(1):60–7. (https://pubmed.ncbi.nlm.nih.gov/22135575/). 22135575 PMC3216201

[16] Rowley MW Agarwal S Seetharam AB Hirsch KS. Real-time ultrasound-guided paracentesis by radiologists: near zero risk of hemorrhage without correction of coagulopathy. J Vasc Interv Radiol. (2019) 30(2):259–264. 10.1016/j.jvir.2018.11.001 30717961

[17] Wubben BM Dandashi J Rizvi O Adhikari S. Emergency physician performed ultrasound-guided abdominal paracentesis: a retrospective analysis. POCUS J. (2024) 9(1):75–79. 10.24908/pocus.v9i1.16668 38681156 PMC11044928

[18] Atkins K Schneider A Charles A. Splenic salvage: is there a role for splenorrhaphy in the management of adult splenic trauma? Am Surg. (2023) 89(12):5599–608. 10.1177/00031348231156760 36878857 PMC11702913

[19] Harfouche MN Dhillon NK Feliciano DV. Update on nonoperative management of the injured spleen. Am Surg. (2022) 88(11):2649–55. 10.1177/00031348221114025 35816431

